# Contact Electrification of Individual Water Microdroplets Inside Mass Spectrometers Leads to Extensive Oxidation or Reduction Irrespective of Initial Droplet Charge or Polarity

**DOI:** 10.1021/acscentsci.6c00443

**Published:** 2026-09-07

**Authors:** Matthew S. McPartlan, Casey J. Chen, Evan R. Williams

**Affiliations:** Department of Chemistry, 1438 University of California, Berkeley, California 94720-1460, United States

## Abstract

Charged water microdroplets that are aerodynamically accelerated upon introduction into an atmospheric sampling mass spectrometer interface can undergo glancing collisions that lead to surprising electrochemical processes. A custom charge detection instrument with a 24-element detector array is used to determine the outcomes of glancing collisions of individual charged water microdroplets with stainless-steel surfaces over a well-defined and narrow angle of ∼0.15° ± 0.03°. Water droplets entering the high-vacuum region with angles of <0.11° pass through the detector unimpeded and have kinetic energies in the MeV range depending on droplet size due to aerodynamic acceleration alone. Droplets that have glancing surface collisions undergo extensive oxidation or reduction through the removal or addition of up to tens of thousands of electrons per collision, independent of their initial charge or polarity, although some ion transfer may also play a role. The propensity for these two electrochemical processes appears stochastic, although more highly charged droplets appear to undergo charge reduction more readily. These results clearly demonstrate that highly energetic electrochemical processes of any type can occur within charged or uncharged aqueous microdroplets of any polarity that are introduced into mass spectrometers and may explain the observation of some unusual ions that have been reported in some microdroplet reaction studies.

## Graphical abstract

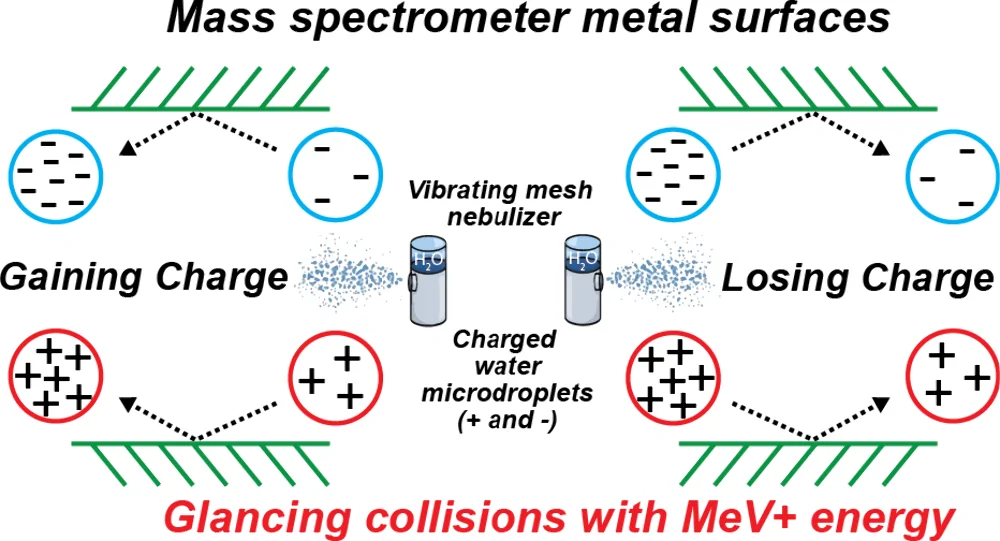

## Introduction

Chemistry in water microdroplets has attracted intense interest following reports of reaction rate enhancements exceeding up to 6 orders of magnitude relative to bulk solution,^1,2^ along with observations of unexpected chemical reactions.^3–10^ These effects have been attributed to properties of the air–water interface,^11,12^ including partial solvation,^13,14^ strong interfacial electric fields,^15–17^ and interactions with gaseous species.^18,19^ However, most (but not all) experiments reporting accelerated microdroplet chemistry involve evaporating droplets, where reactant concentration alone can account for rate enhancements of up to 7 orders of magnitude under some conditions,^20,21^ making it important to consider effects of reagent concentration in a mechanistic understanding of potential interfacial effects.

A variety of spontaneous reactions attributed to the unique interfacial properties of water microdroplets have been reported (see Supporting Information, Section S1).^22,23^ One of the most striking claims in water microdroplet chemistry is the spontaneous formation of micromolar concentrations of hydrogen peroxide proposed to arise from abundant hydroxyl radical (OH·) generation driven by high intrinsic interfacial electric fields.^24,25^ Evidence for spontaneous hydroxyl radical formation in unactivated water droplets in the form of ions speculated to be reaction products has been reported.^25,26^ However, many others report that these ions instead originate from contamination.^27–32^ It should be noted that hydroxyl radicals, hydrogen peroxide, or other reactive species may be generated when water or water droplets are activated by ultrasound,^33–35^ contact electrification from liquid–solid or droplet-solid interactions,^36–38^ electrical discharge^39,40^ including discharges that can occur with electrospray,^32,40,41^ and through interactions between positively and negatively charged droplets.^12,42,43^ However, when dissolved oxygen and ozone are rigorously excluded in water microdroplet studies where activation is minimized, hydrogen peroxide is not detected in neutral or positively charged water microdroplets when probed using techniques that have detection limits of ∼50−60 nM.^19,36,37,44^ This is consistent with water being stable at the air–water interface of microdroplets.

In striking contrast to neutral or positively charged aqueous microdroplets, micromolar concentrations of hydrogen peroxide are produced in negatively charged water microdroplets generated by electrospray ionization.^44^ In negative electrospray, OH^–^ is electrochemically generated in the emitter to produce droplets with considerable excess OH^–^ as the net charge carrier. As droplets evaporate and approach the Rayleigh limit, fission or ion/electron emission become favorable processes that are driven by the Coulombic repulsion between excess charges.^45^ Electron emission either directly from droplets^45^ or upon droplet surface contact leads to OH· formation and subsequent hydrogen peroxide production that is directly related to electrospray current.^44^ This process is activated and requires energy far exceeding the thermodynamic minimum (by ∼500×). Energy input and activation are often overlooked in studies of microdroplet chemistry, especially those relying on atmospheric pressure sampling mass spectrometers used for detection leading to claims of “eco-friendly” or “green” chemistry.^5,46^ Although water is an environmentally friendly reagent, a recent analysis of the formation of nitric acid and ammonia with water microdroplets found that the resulting products were ∼15,700× and ∼269,000× more costly to produce, respectively, than standard methods.^47^ Energy input is required to generate microdroplets from liquid water, whether through compressed gases used in pneumatic nebulization or electrical power applied during electrospray or ultrasonic nebulization.

Additional activation can occur within a mass spectrometer itself. Recent charge detection mass spectrometry measurements show that neutral water droplets introduced through stainless-steel interfaces can undergo contact electrification during glancing collisions with metal surfaces.^38^ This leads to extensive oxidation, producing droplets carrying thousands to hundreds of thousands of charges. Oxidation of water by contact electrification produces OH· that leads to hydrogen peroxide as well as the potential for direct oxidation of other species near the droplet surface. The driving force for this nonspontaneous oxidation chemistry is the high kinetic energy imparted to droplets (MeV+) by aerodynamic acceleration through the instrument interface as droplets transit from atmospheric pressure into vacuum. Both contact electrification and mechanical breakup of water can produce positively and negatively charged microdroplets. The OH· and ultimately hydrogen peroxide generated by these mechanisms for negatively charged droplets may account for the observation of reactive oxygen species in droplets produced by pneumatic nebulization.^3,8,24^ Gaseous analyte molecules can also incorporate into neutral microdroplets, yielding electrospray-like mass spectra upon contact electrification inside the mass spectrometer.^38^ Electrospray-like spectra are also produced when protein-containing solvents are directly introduced into heated capillaries of mass spectrometers,^48,49^ a process that is likely also driven by contact electrification.^38^ Contact electrification and liquid–solid interactions that occur outside of a mass spectrometer in devices such as triboelectric nanogenerators have been reported^50^ and can lead to reactive species.^51,52^

Directly probing droplet-surface interactions inside a mass spectrometer, especially in small-diameter stainless-steel capillaries, has remained experimentally inaccessible. Here, we overcome this limitation by using a charge detection instrument uniquely capable of resolving changes in droplet charge and velocity arising from glancing collisions of individual water microdroplets with metal surfaces. Using water droplets generated by a vibrating-mesh nebulizer, we show that single collisions can either increase or decrease charge for droplets of either polarity. These observations demonstrate that contact electrification inside of mass spectrometers can drive both oxidative and reductive processes in microdroplets independent of their initial charge or polarity. These results indicate that many low-abundance ions previously attributed to spontaneous electric-field driven chemistry in water microdroplets may instead be generated by droplet-surface collisions, underscoring the need to account explicitly for droplet activation and instrument-induced processes in microdroplet chemistry studies.

## Results and Discussion

### Charge Detection Array and Droplet Collision Angles

A schematic diagram of the instrument used in these experiments is shown in Figure 1. This instrument has an array detector consisting of 24 stainless-steel tubes.^38^ Every other tube is connected to form two detection channels. These channels are differentially amplified to produce a time-domain signal of a charged droplet passing through the detector array. Importantly, *all ion optics prior to the array detection system were grounded to create a field-free path through which the droplets pass*. Thus, the droplet trajectories are determined entirely by aerodynamic effects as the ions transit from atmospheric pressure into the first vacuum stage of the mass spectrometer. While this expansion primarily accelerates droplets along the axis of the instrument, the absence of any ion focusing elements allows droplets to spread out into a cone-shaped distribution as they exit the capillary interface. For a droplet to reach the detector, it must exit the ion funnel region with an angle of less than 0.18° relative to the centerline of the instrument or it will not pass through the grounded shield tube that is located immediately in front of the detector array (Figure 1, Figure S1). If a droplet has an angle that is less than 0.11° from the centerline of the instrument, it will pass through all 24 elements of the detector array without surface collisions. Thus, droplets traveling at intermediate angles between 0.11° and 0.18° off the centerline will make glancing contact with at least one element in the detector array.

**Figure 1:**
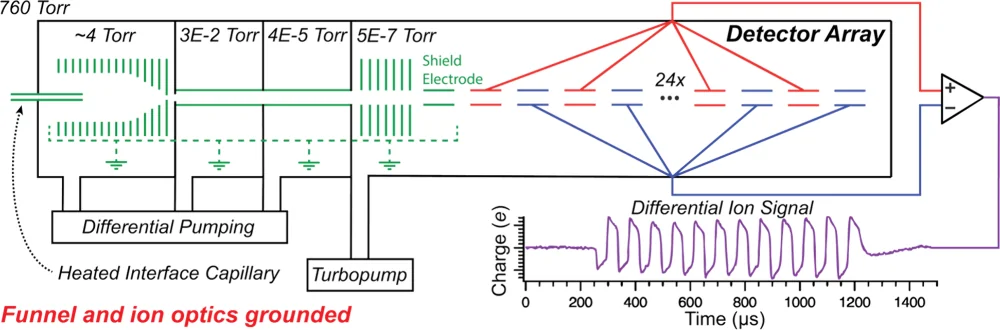
Schematic diagram (not drawn to scale) of the Berkeley linear array charge detection mass spectrometer operated as a polarity independent charge detection instrument in which all ion optical elements are grounded. Droplets enter the instrument through an atmospheric sampling interface and pass through three differentially pumped vacuum chambers before passing through a 24-tube charge detection array. Water microdroplets entering from atmospheric pressure through a stainless-steel capillary interface do not experience any electric fields throughout the entire transit time through the instrument, including the charge array detector.

### Droplet Charge Measurements

A typical droplet that passes straight through all 24 of the detector tubes of the charge detection instrument induces a symmetric and roughly square wave signal that has 24 pulses of alternating polarity. The droplet polarity can be determined from the amplitude of the initial pulse. A positive initial pulse, shown in Figure 2a, is induced by a positively charged droplet whereas a negative initial pulse, shown in Figure 2d, is induced by a negatively charged droplet. The overall charge on the droplet is determined from the peak-to-peak amplitude of two adjacent cycles. The first and last cycles have greater variations in amplitude and are not considered in our analysis. The remaining cycles provide 22 measurements of droplet charge for a droplet that transits all tubes. The net charge of a droplet is obtained from the mean peak-to-peak amplitude of these 22 cycles. For the positively charged droplet shown in Figure 2a, the net charge is +11,920 *e* with a charge uncertainty of ±260 *e*. This charge measurement was stable (Figure 2c, pink line) as the ion transited the detector array, showing that the ion did not undergo a measurable change in charge. The velocity of this ion is determined by computing the derivative of the induced signal (Figure 2b). The peaks in the derivative correspond to when a droplet is between detector tubes. The time interval between these peaks, together with the length of each detector tube and the space between tubes, provides a measurement of droplet velocity as it passes through the detector array. The average velocity of this positive droplet was 388 ± 15 m/s and the velocity was unchanged as it transited the array (Figure 2c, teal data). These results are consistent with a droplet that passed through the detector array without interacting with the stainless-steel detector tubes (other than by inducing an image charge current that is detected).

**Figure 2:**
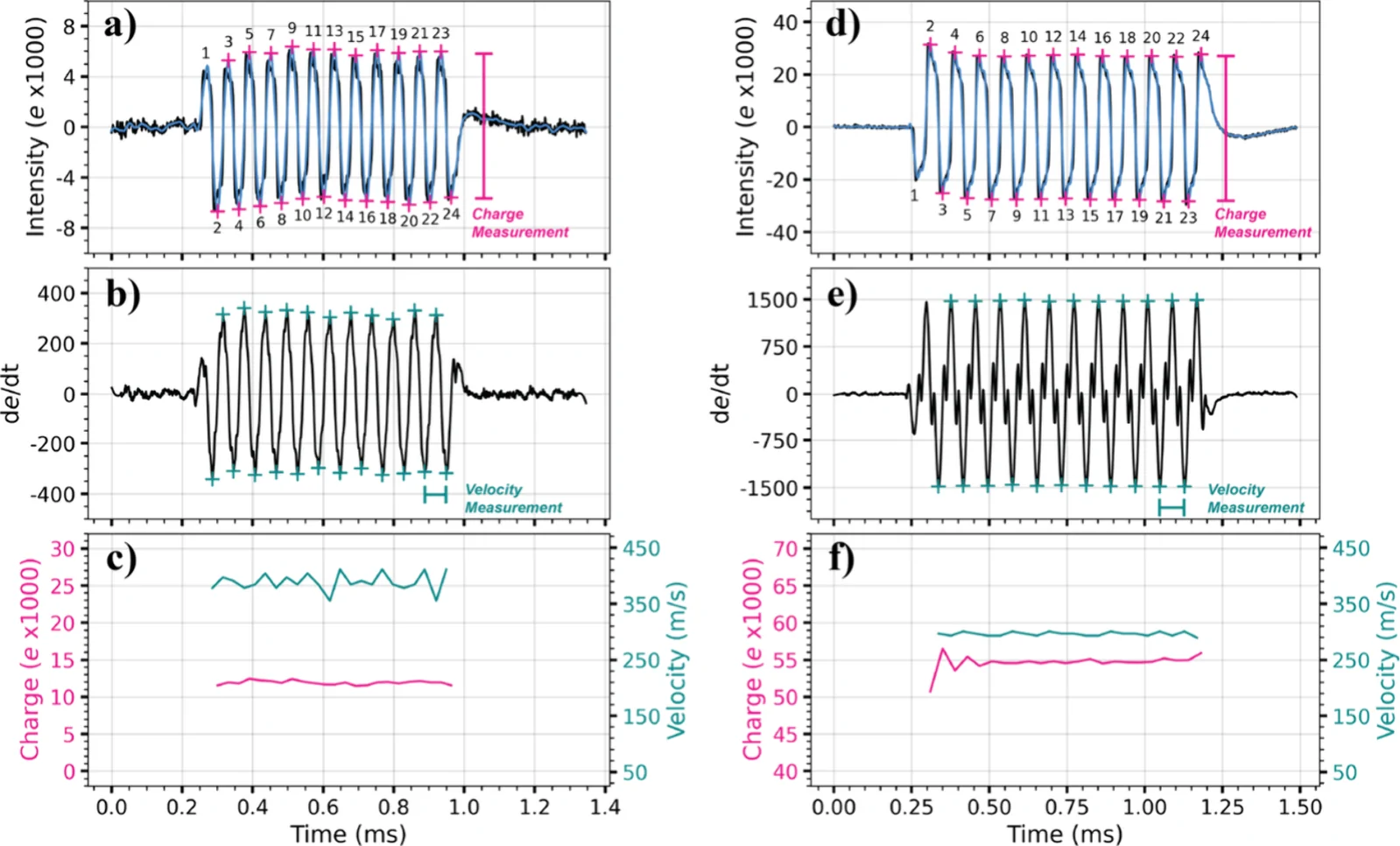
Examples of ions transiting all 24 tubes of the detector array without a measurable change in charge or velocity. The polarity of each droplet is determined by the directionality of the initial pulse. (a) raw (black) and smoothed (Savitsky-Golay, 101-point window) signal (blue) of a positively charged droplet with +11,920 *e*, identified by a positive initial pulse, transits all 24 tubes; (b) first derivative of the signal in (a) where the marked points are used to determine the velocity; (c) droplet charge (in pink) and velocity (in teal) determined from the amplitudes of the signals in (a) and time between peaks in (b), respectively; (d), (e) and (f), the same respective information as in (a), (b), (c) for a negatively charged droplet with −54,840 *e*, identified by a negative initial pulse.

A signal for a significantly more highly charged negative droplet is shown in Figure 2d. This droplet has a net charge of −54,840 ± 1,030 *e* and a velocity of 296 ± 3 m/s (Figure 2f, pink and teal data, respectively). Neither the charge nor the velocity changed measurably as the droplet passed through the array. The constant charge and velocity indicate that this droplet also passed through the detector without colliding with any detector tube. To quantify uncertainty in a typical charge measurement, eight additional droplets that passed through all 24 detector tubes with no detectable collisions were characterized (details in Supporting Information). These droplets ranged in charge from approximately −4,000 *e* to +13,000 *e* with charge uncertainties between ± 219 *e* (2,590 *e* droplet) and ± 471 *e* (10,010 *e* droplet) with a mean of ± 287 *e*. There does not appear to be a correlation between droplet charge and measurement uncertainty (Figure S2). The higher uncertainty for the droplet shown in Figure 2d is caused by a detector ‘settling’ effect in the first 3−4 tubes. This effect is present in every induced signal but is more significant in signals induced by droplets with very high charge (>∼20,000 *e*). If these tubes are not included in our analysis, the charge of this droplet measured starting with tube 5 is 54,730 ± 235 *e*. These results suggest that this detector array is capable of reliably discerning charge changes with a magnitude greater than ∼300 *e*, especially in cases where a charge change occurs after detector tube 4.

### Contact Electrification of Positively Charged Droplets

Droplets that exit the interface capillary with an angle between 0.11° and 0.18° off-center will pass through the detector shielding tube but will collide with the surface of a detector tube before reaching the end of the array. The array geometry ensures that these glancing collisions occur over a narrow range of angles of just 0.07°. Some of these droplets pass through the entire array after a collision. Collisions with the detector tube wall can produce a significant change in the signal, and the independent nature of each charge measurement makes it possible to monitor the droplet charge and velocity over the course of each interaction that occurs within the detector array.

An example of the signal for one such positive ion is shown in Figure 3a, where a relatively low-charged droplet enters the detector array with an initial charge of +2,860 ± 140 *e*. The amplitude of the induced signal increases suddenly after detector tube 11, corresponding to a charge increase of nearly +2,000 *e* to a total droplet charge of +4,770 ± 310 *e*, ∼67% higher than its initial charge. Despite this large increase in charge, there was no significant change in velocity. The droplet was moving at 330 ± 24 m/s before it passed through detector tube 11 and 324 ± 24 m/s after passing through this detector tube. The large increase in charge must be caused by a droplet-surface interaction that resulted in the stripping of ∼1,900 negative charges.

**Figure 3:**
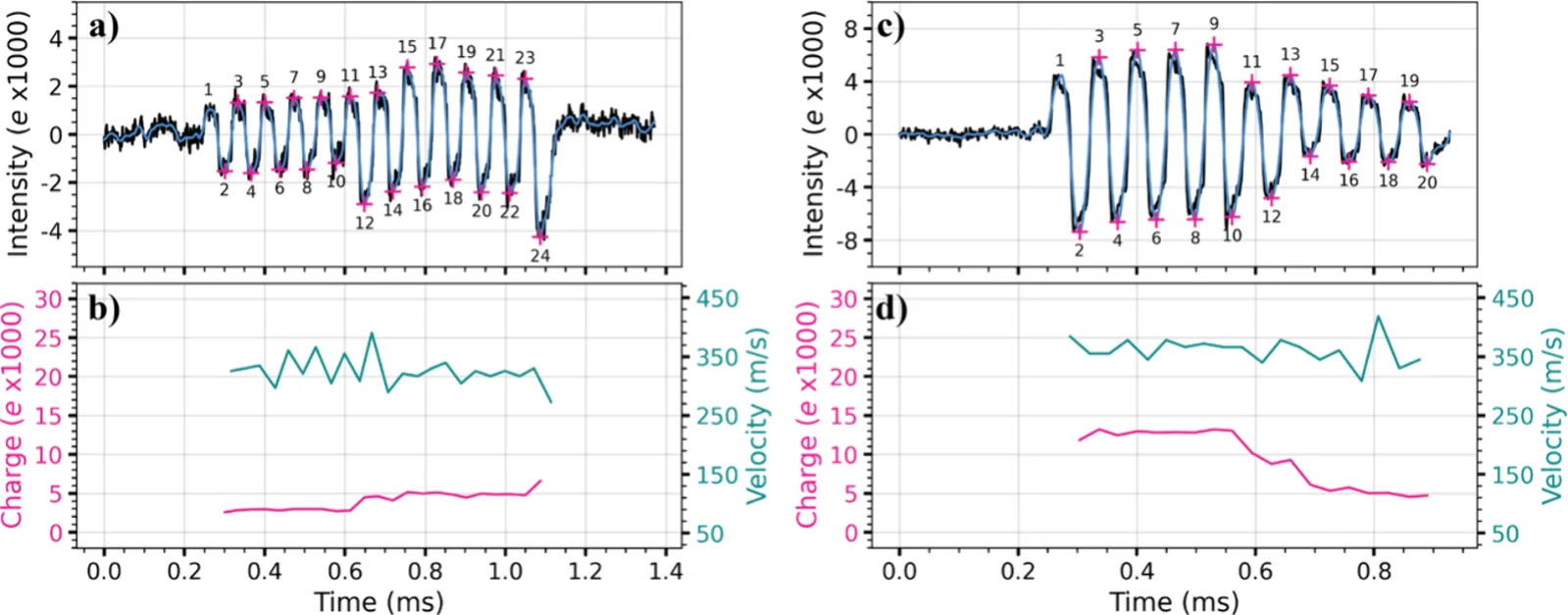
Two examples of positively charged droplets interacting with one or more of the array detector tubes. A positive droplet (a) with an initial charge of +2,860 *e* collides with metal detector tube 11 which increases the charge to +4,770 *e*; (b) the droplet charge (in pink) and the velocity (in teal) as it transits through the array; (c) a positively charged droplet with an initial charge of +12,800 *e* collides with detector tube 10, reducing its charge to +9,030 *e*. A second interaction occurring shortly after with tube 12 further reduces the charge to +5,210 *e*; (d) the droplet charge (in pink) and the velocity (in teal) as it transits through the array.

High-energy glancing collisions between initially charged droplets and a metal surface can produce a variety of surprising outcomes. A different positively charged droplet, shown in Figure 3c, was initially charged at +12,910 ± 250 *e* and had a velocity of 365 ± 12 m/s. This droplet underwent an initial interaction with detector tube 10 that resulted in a *reduction* of charge to +9,420 ± 720 *e*. Shortly after this initial interaction, a second interaction with detector tube 12 further reduced the charge to a final value of +5,210 ± 560 *e*. The velocity does not measurably change (final velocity of 353 ± 36 m/s) over the course of these two interactions (Figure 3d). This indicates that any change in droplet kinetic energy that occurred as a result of these collisions is not measurable in these experiments. These results provide evidence that a positively charged water droplet can either gain or lose positive charge when it interacts with the stainless-steel surface of the detector electrodes.

In prior neutral droplet contact electrification experiments, positively charged droplets were primarily produced.^38^ A small fraction of negative droplets was also produced, and their formation was attributed to mechanical breakup of larger droplets or contact electrification. While mechanical breakup cannot be ruled out, the direct measurement of charge loss here during very similar interactions in the detector array itself provides support for the hypothesis that negative droplets may also be formed from neutral water droplets by contact electrification during droplet-metal interactions inside the interface capillary.

One possibility for the different behavior of initially charged droplets with respect to adding or reducing positive charge is the magnitude of droplet charge relative to the Rayleigh limit. In these experiments, the masses of the droplets are not measured directly because all ion optics are grounded. However, the droplet velocity does provide an indication of the relative droplet size. Larger droplets in a beam of streaming gas move more slowly than smaller droplets in the same gas stream, i.e., they have a higher slip velocities.^38,53,54^ The droplet shown in Figure 3c had a higher velocity than the droplet in Figure 3a (365 and 330 m/s, respectively), indicating that the former droplet (Figure 3c) is much smaller. Despite its smaller size, it had an initial charge of nearly +13,000 *e* compared to +3,000 *e* for the larger droplet shown in the Figure 3a. Thus, a smaller, faster, droplet that has significantly more charges could be more prone to charge reduction during an interaction than a larger, less highly charged droplet. For a given charge, a smaller droplet has more Coulombic repulsion and is charged more closely to the Rayleigh instability limit. Such a droplet may be more likely to donate charge than a larger droplet charged well below the Rayleigh limit.

Ion-surface collisions have been used to characterize structures of large macromolecular complexes, a method referred to as surface-induced dissociation (SID).^55–58^ Collisions in SID devices typically occur at much higher angles^56^ than the droplet collisions occurring in the detector array presented here (∼0.15° ± 0.03°). Reduction of charge often occurs in SID for small ions without a surface coating.^56,57^ In contrast to the extensive droplet oxidation or reduction observed here with glancing collisions, little change in charge occurs in SID for larger macromolecular complexes with or without surface coatings.^55,58^ The extreme glancing collision angle in the current experiments has the obvious effect of converting significantly less kinetic energy into droplet internal energy than would be expected in an equivalent SID collision.

A Weber number, *We*, has been used to predict whether a droplet will fragment (eq 1),^59–61^

1 $$We=\frac{\rho v^{2}R}{\sigma }$$

where ρ is the fluid density, *v* is the droplet velocity, *R* is the droplet radius, and σ is the fluid surface tension. A value of *We* ≫ 1 indicates a kinetic energy significantly greater than what can be stored as surface energy during deformation, thus indicating a droplet will likely dissociate upon a collision. The droplet shown in Figure 3a initially has +2,860 *e* and is initially moving along the axis of the instrument at 330 m/s. If charged at 30% of the Rayleigh limit and colliding with the detector array at the maximum collision angle of 0.18 degrees, this droplet has a diameter of ∼350 nm, a radial collision velocity of ∼1 m/s, and Weber number of ∼0.003. Since *We* ≪ 1, this droplet is not expected to fragment from this collision.^61^ Results for the much larger and hence higher kinetic energy droplet shown in Figure 2d (−54,840 *e*, 296 m/s axial velocity) would indicate a maximum Weber number of ∼0.015. Thus, even the highest kinetic energy droplets observed here are not predicted to fragment during these glancing collisions. We note that the detector electrode surfaces were polished and cleaned prior to installation but are not molecularly smooth, nor has the nature of the surface been well characterized. Thus, these calculations are only a guide. While it is not possible to rule out fragmentation from these calculations or from sheer forces that are not included in these calculations, there is no evidence for droplet fragmentation apparent in the experimental data. Fragmentation would be reflected by a broadening of the signal response between detector tubes due to Coulombic repulsion between, and separation of, charged progeny droplets formed by fragmentation.

### Charge Transfer by Electrons and Ions

Charge transfer between a droplet and the detector tube surface could occur either by transfer of electrons or ions in the droplet. The concentration of H_3_O^+^ and OH^–^ in a neutral aqueous droplet at pH 7.0 is roughly 10^–7^ M, neglecting effects of the interface on the autoionization of water. For a 500 nm water droplet, this concentration corresponds to four H_3_O^+^ and four OH^–^ ions within the droplet. Contact electrification of neutral droplets that occurs via glancing collisions in a stainless-steel capillary of electrospray interfaces can lead to positively charged droplets with many thousands of charges in this droplet size range.^38^ Thus, thousands of times more negative charges are removed from neutral droplets than the number of negative ions in the droplet, even considering alternative anions from adventitious salts. Moreover, these charge carriers are dispersed in a neutral droplet, but the transfer of ions would be more localized to ions that are at the surface. For water droplets with a net positive charge, the OH^–^ concentration is even lower, further favoring an electron transfer mechanism for increasing droplet charge. Transfer of existing ions inside the droplet cannot account for the large charge increases observed for neutral droplets or for the charged droplet collisions investigated here. It is possible that autoionization of water is enhanced via shearing or other forces that occur during these glancing surface collisions and that some of the charge change that occurs may be due to the transfer of ions to the surface. This would require the charge carrying species to be generated and subsequently lost with a large charge imbalance over the relatively brief time scale of the collision (up to ∼30 μs). The large asymmetry in production of positive droplets from neutral water droplets in contact electrification inside heated metal capillary interfaces is inconsistent with significant charge transfer originating from the transfer of ions.^38^ Thus, although ion transfer is plausible, it is likely that most of the charge transfer occurs via electrons. In contrast, ion transfer may play a greater role when reduction of charge occurs in these collisions because there is a net charge carrier located near the surface, although it is likely that electron transfer is dominant when the change in net charge is significant.

### Contact Electrification of Negatively Charged Droplets

Remarkably, contact electrification that can induce either oxidation or reduction of positively charged droplets can induce either oxidation or reduction of negatively charged droplets as well. Figure 4a shows results for a droplet (initial charge of −5,405 ± 372 *e*, initial velocity of 323 ± 19 m/s) that interacted with detector tube 13. This interaction more than doubles the initial charge on this droplet to a final value of −12,941 ± 794 *e*. A different negatively charged droplet shown in Figure 4c enters the detector array with −9,260 ± 286 *e* charges and a velocity of 320 ± 15 m/s. This droplet had a slightly higher charge but similar velocity to the microdroplet shown in Figure 4a. However, an interaction with detector tube 11 led to a *decrease* in droplet charge. Neither droplet exhibits a significant change in velocity over the course of these interactions, with final velocities of 318 ± 13 m/s and 315 ± 15 m/s for droplet 4*a* and droplet 4*b*, respectively. While addition of thousands of charges to an already charged droplet almost certainly had a high energetic cost, it was not sufficiently large to be measured in these experiments.

**Figure 4:**
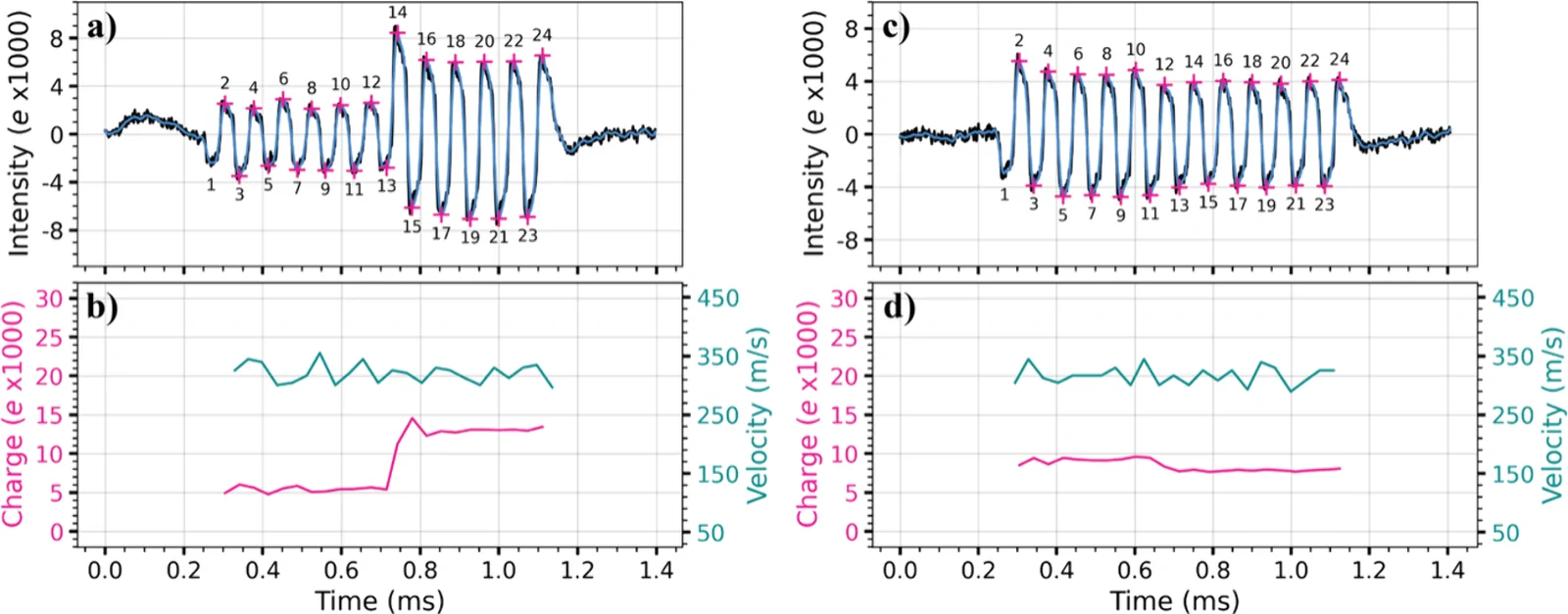
Two examples of negatively charged droplets interacting with one or more of the array detector tubes. A negative droplet (a) with an initial charge of −5,405 *e* collides with metal detector tube 14, which increases the charge to −12,941 *e*; (b) the droplet charge (in pink) and the velocity (in teal) as it transits through the array; (c) a negatively charged droplet with an initial charge of −9,260 *e* collided with detector tube 11, reducing its charge to −7,845 *e*; and (d) the droplet charge (in pink) and the velocity (in teal) as it transits through the array.

### Stochastic Nature of Charge Transfer

All examples of droplet-surface collisions discussed thus far correspond to monotonic addition or reduction of a droplet’s charge, but a single droplet can have multiple, independent interactions with subsequent detector tubes that each have different outcomes. A positive droplet that exhibits this mixed behavior is shown in Figure 5. The droplet entered the detector array with a charge of +8,870 ± 295 *e*. An interaction with detector tube 12 significantly reduced the droplet’s charge to +4,160 ± 740 *e*. A second interaction with detector tube 19 then led to an increase in the droplet’s charge to a final value of +5,590 ± 110 *e*. Thus, the outcome of one interaction does not predetermine the outcome of subsequent interactions. While the initial charge of the droplet relative to the Rayleigh limit appears to be a factor in whether a droplet is oxidized or reduced through contact electrification in these glancing collisions with stainless-steel surfaces, there does not appear to be any other characteristic of a water droplet that would lead us to predict whether it will gain or lose charge, either positive or negative.

**Figure 5:**
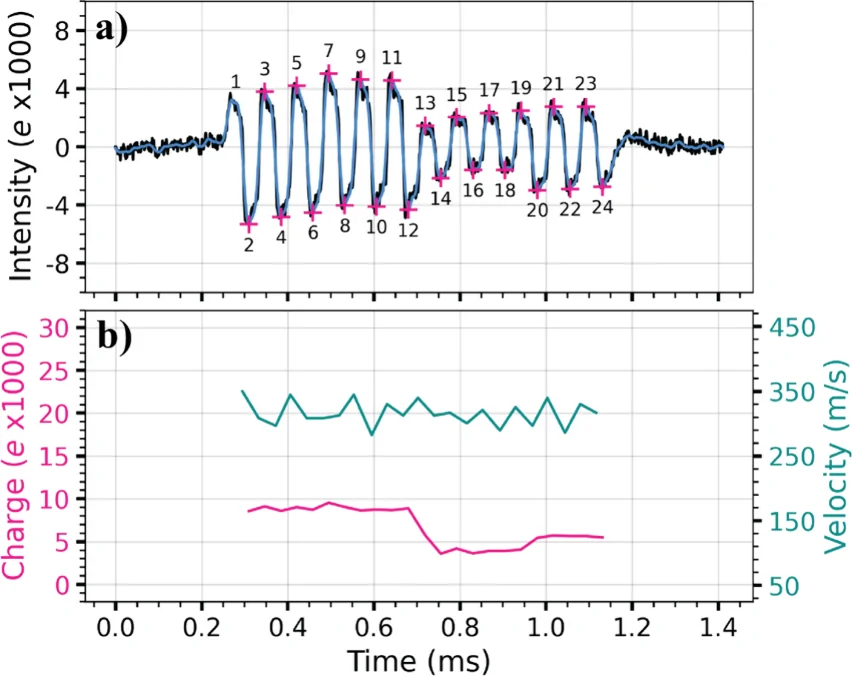
A positively charged droplet (a) with an initial charge +8,870 *e* that had two collisions with different detector tubes resulting in electrochemical reduction then oxidation. The first interaction occurred in detector tube 12 and led to a charge reduction to +4,160 *e*. The second interaction occurred within tube 19 and led to a charge increase to +5,590 *e*; (b) the velocity (teal data) remains constant throughout these interactions (318 ± 21 m/s initial, 314 ± 22 m/s final) and the change in charge is shown in pink.

### Interactions with High-Energy Transfer

In contrast to the above examples where a significant change in charge was not accompanied by a measurable change in velocity, there are droplets for which significant energy transfer occurs upon collisions with a surface. Figure 6a shows an example of a negative droplet undergoing an interaction that increases the charge from −37,830 *e* to −60,170 *e* and decreases the velocity from 242 to 145 m/s. Surprisingly, this is followed by a series of interactions that gradually reduce the velocity of the droplet to ∼100 m/s with a nearly inconsequential reduction in charge to −59,170 *e*. This result indicates that substantial energy transfer can occur without subsequent oxidation or reduction of the droplet. Thus, interactions can affect droplet charge, droplet velocity, or a combination of the two. A significant change in droplet charge is the most consistently observable indication that a droplet-surface interaction has occurred, but a significant transfer of charge does not occur with every glancing collision. It is possible that many interactions do not transfer a detectible number of charges and that our results are somewhat biased toward the more extreme cases.

**Figure 6:**
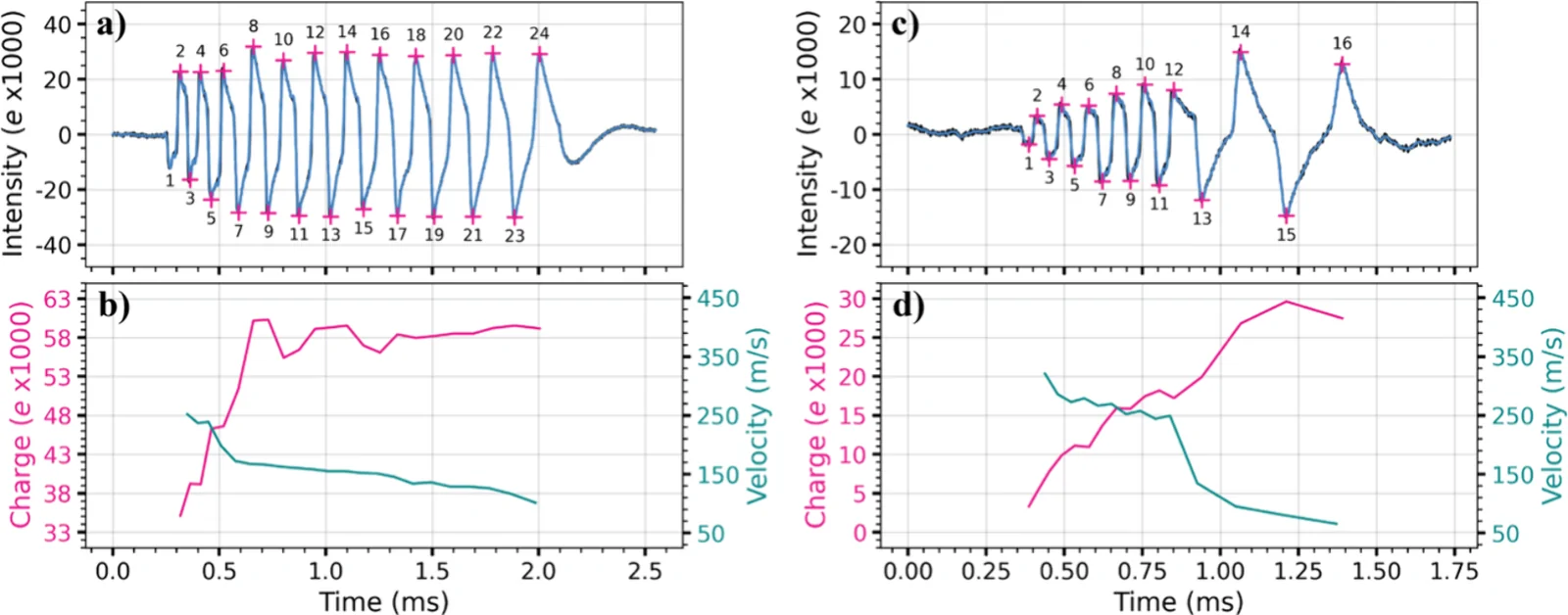
Examples of multiple collisions accompanied by large reductions in kinetic energy for (a) a negatively charged droplet that underwent a single interaction that increases the charge from −37,830 *e* to −56,850 *e* (b, pink data) and reduces the velocity from 242 to 145 m/s (b, teal data). A negatively charged droplet (c) that underwent multiple collisions in which its charge steadily increased from an initial charge of −3,310 *e* to a final charge of −29,630 *e*. The charge (d, pink data) and velocity (d, teal data) show that the changes in charge are accompanied by decreases in droplet velocity with an initial velocity of 321 m/s and a final velocity of 66 m/s over the course of these multiple interactions. Ultimately, these collisions caused this droplet to be lost from the detector array after tube 16.

Another unexpected type of droplet-surface collision is shown in Figure 6c. This droplet had an initial charge of −3,310 *e* and velocity of 321 m/s. The signal on each sequential detector tube increased in amplitude with a corresponding decrease in velocity. The charge and velocity at the last tube was −29,630 *e* and 66 m/s, respectively, corresponding to an overall increase in charge by ∼9x and a decrease in velocity of ∼5x. This suggests that this droplet “rolled down” the detector array, interacting with nearly every single detector tube before undergoing a “catastrophic” collision with tube 16 leading to a loss of signal. This result is consistent with a substantial kinetic energy loss driving reactions that would otherwise not occur.

These results also indicate that the initial charge on most of these droplets is substantially below the Rayleigh limit. Mechanical breakup of liquid water can produce droplets with both polarities, but this process is expected to lead to less charging than typically observed with electrospray. Droplet evaporation in vacuum occurs and leads to an increase in charge density. About 8% of the mass of an 82 nm water droplet was lost over a 1 s period during which the droplet was trapped in a CDMS instrument,^62^ a fraction that is typical for a wide range of droplet sizes.^62,63^ The relatively low mass loss in high vacuum is due to evaporative cooling which reduces the effective temperature of the droplets. In the current experiments, droplets are in vacuum for only ∼2–4 ms. The evaporative mass loss inside the instrument that occurs during this time is negligible (<0.1%). A low capillary temperature also reduces droplet evaporation in the higher-pressure region of the instrument interface so that increases in charge density should also be minimized throughout these experiments. Thus, most droplets in the detector array under these conditions are not expected to be charged close to the Rayleigh limit. The relatively low initial charge may be why such significant increases in charge can occur for both positively and negatively charged droplets in these glancing collisions.

### Energetic Effects of Charge Transfer Interactions

Oxidation of positively charged droplets or reduction of negatively charged droplets has an energetic cost because more highly charged droplets are less stable owing to Coulombic repulsion. The energy lost in these collisions can be determined from the reduction in velocity and estimated droplet size. For the droplet shown in Figure 6c,d, if the final charge of −29,630 *e* led to this droplet being charged at the Rayleigh limit prior to its loss from the detector array after tube 16, the initial droplet charge of −3,310 *e* indicates it was charged at ∼11% of the Rayleigh limit prior to the first glancing collision. This extent of charging corresponds to a droplet mass and diameter of 143 GDa and 770 nm, respectively. The initial energy determined from the mass and initial velocity of 321 m/s is 76.5 MeV. The final velocity of 66 m/s prior to droplet loss corresponds to an energy of 3.2 MeV. Thus, 73.3 MeV was lost in collisions corresponding to an average energy of 2.7 keV for each charge that was gained.

For droplets without a measurable change in velocity, the corresponding energy lost per charge gained is much lower. An upper limit can be estimated from the uncertainty in the velocity measurements and assuming initial charging at the Rayleigh limit. If initially charged at the Rayleigh limit, the positive droplet shown in Figure 3a (initial charge of +2,860 ± 140 *e*, final charge of +4,770 ± 310 *e*), would have an approximate mass of 1.3 GDa (∼160 nm diameter) and a corresponding kinetic energy of ∼703,000 eV. The uncertainty in the measured velocity for this droplet (∼7%) corresponds to ∼102,000 eV in total or ∼52 eV per charge gained. Roughly 0.004% of the constituent water molecules would therefore be ionized in this process. This is an upper limit because the initial droplet charging was almost certainly well below the Rayleigh limit. If charged at 30% of the Rayleigh limit, this droplet would have been ∼14.4 GDa (∼360 nm in diameter) with a kinetic energy of ∼6.7 MeV. The uncertainty in the measured velocity for this droplet (∼7%) corresponds to ∼1.14 MeV or ∼596 eV per charge gained.

## Conclusions

This study demonstrates that glancing-angle collisions between charged water microdroplets and stainless-steel surfaces trigger significant and unexpected charge transfer processes inside of mass spectrometers. By precisely controlling collision angles between 0.11° and 0.18°, we observe that water microdroplets of either polarity can undergo both oxidation and reduction through the transfer of up to tens of thousands of electrons. These results indicate that the formation of both positively and negatively charged water droplets from neutral precursors within mass spectrometer interface capillaries, as previously reported,^38^ can occur via contact electrification at glancing angles. While reduction of positive ions has been observed previously in ion-surface collisions,^56,57^ we are not aware of any report where oxidation has been reported. An even more surprising observation in this work is that ion-surface collisions can add up to tens of thousands of electrons to water microdroplets that are already negatively charged. The high kinetic energy (MeV+) imparted to the droplets by aerodynamic acceleration in the transition from atmospheric pressure into vacuum provides the energy needed to drive the endothermic process of increasing droplet charge. Importantly, these results show that high-energy oxidation or reduction of water microdroplets can result from droplet-surface interactions that can occur in any atmospheric sampling mass spectrometer. Such glancing collisions occur in instruments with metal interface capillaries^38^ and can occur with other downstream ion optical elements. Although initial microdroplet charge density may influence whether oxidation or reduction occurs, the specific result of an individual droplet-surface collision is unpredictable. Both oxidation and reduction can occur for the same microdroplet in sequential collisions indicating the stochastic nature of the contact electrification processes.

Unlike the charge detection mass spectrometer used in this study, most commercial instruments do not have a direct line of sight between the atmospheric sampling source and the detector region. Off-axis geometries are designed to prevent the beam of neutral gas molecules and surviving microdroplets that are generated by the pressure difference in the interface from interfering with detection. In such instruments, neutral droplets surviving the interface vacuum transition inevitably impinge on internal surfaces, most commonly metal. Charged droplets behave similarly. Because of their high kinetic energy and extreme *m*/*z* that is outside the range of most radio frequency (RF) confinement devices, they undergo minimal deflection with electric fields and standard RF ion optics. For example, a water droplet with 1 μm diameter charged at 50% of the Rayleigh limit traveling at ∼210 m/s has a *m*/*z* = 1.4 × 10^7^ and a kinetic energy of ∼72 MeV, values well beyond the capabilities of commercial instruments to control. These droplet-surface collisions could occur over a wide range of angles leading to a significant fraction of the MeV+ kinetic energy being deposited into the microdroplets. High-energy impacts of this nature can induce extensive breakup of the microdroplet and direct ionization of the solvent or analyte molecules. They can also lead to desorption of species previously deposited on mass spectrometer surfaces.^21,38^ Consequently, contact electrification observed here at glancing angles likely occurs in any mass spectrometer where droplets, whether neutral or charged, are incompletely desolvated. Given the rapid increase in research on microdroplet reactions and the widespread use of mass spectrometers to analyze products, the extensive redox chemistry occurring upon microdroplet surface collision must be considered.

## Methods

### Charge Detection Mass Spectrometer

A custom designed and built mass spectrometer that has a linear sequential pass detector array was used to measure droplet charge and velocity.^38^ A schematic diagram of this instrument is shown in Figure 1. The atmospheric sampling interface is similar to those in commercial instruments that introduce ions from atmospheric pressure into the mass spectrometer through a metal capillary. A mixture of positively and negatively charged water droplets was produced using a vibrating mesh nebulizer (Meowyn, purchased from Amazon) that was positioned roughly 10 cm away from the instrument inlet. The nebulizer and reservoir were purged with Milli-Q water for a minimum of 1 h prior to use. No electrical potentials were applied to the water in these experiments.

A small fraction of the total number of droplets produced enter the mass spectrometer through a stainless-steel capillary (11.4 cm long, 0.5 mm ID) heated to 30 °C. Droplets then enter an ion funnel region (∼4 Torr) followed by two subsequent differentially pumped quadrupole regions and a series of stainless-steel tubes before passing into the detector region (∼5 × 10^–7^ Torr). *In these experiments, all ion optics were grounded so that droplets transiting the instrument do not experience any forces due to electric or magnetic fields*.

Surviving droplets then enter the linear array detector, which consists of 24 tubes (2.54 mm inner diameter, 10.16 mm length, 304 stainless-steel) that are positioned 1.53 mm apart and are connected to form two channels. Detector tubes were polished and cleaned with methanol prior to assembly. The inner surfaces of the detector tubes are not well characterized and are likely covered with a hydrocarbon layer originating from oil from mechanical pumps that back the turbopumps as well as contaminants from laboratory air that is continuously introduced through the atmospheric sampling interface. Tubes 1, 3, 5, etc. make up channel **A** while tubes 2, 4, 6, etc., make up channel **B**. An ion passing through the detector induces a series of pulses as it passes through each tube, 12 from channel **A** and 12 from channel **B**. These pulses are differentially amplified to produce an ion signal that is approximately square wave shaped. The peak-to-peak amplitude of each cycle is proportional to charge and was calibrated using a 2 pF test capacitor connected to an external signal equivalent to 60,000 charges. This calibration was verified by comparison to signals of a polystyrene bead standard measured on an accurate electrostatic cone trap charge detection mass spectrometer.^64–66^ Droplet velocities were determined by identifying peaks in the first derivative of the induced ion signal and measuring the time between peaks. This corresponds to the time required for an individual droplet to transit the sum of the distance of a single tube and the space between two tubes. No unusual or unexpected safety hazards were encountered during these experiments.

## Supplementary Material



## Notes

Additional references and context for this work, an image and description detailing ion collision trajectories, an image and accompanying description showing performance of instrument charge measurements (PDF)
