# Supplementary material for: Contact Electrification of Individual Water Microdroplets Inside Mass Spectrometers Leads to Extensive Oxidation or Reduction Irrespective of Initial Droplet Charge or Polarity
Source: ACS Cent Sci. 2026 Sep 7;12(9):1338–47. doi: 10.1021/acscentsci.6c00443 (PMC13614038; doi:10.1021/acscentsci.6c00443)
Supplement: Supplementary file 1 [file oc6c00443_si_001.pdf]

Supporting Information for

Contact Electrification of Individual Water Microdroplets Inside Mass Spectrometers Leads to Extensive Oxidation or Reduction Irrespective of Initial Droplet Charge or Polarity

Matthew S. McPartlan, Casey J. Chen, and Evan R. Williams*

Department of Chemistry, University of California, Berkeley, California 94720-1460, United States

*To whom correspondence should be addressed

Email: erw@berkeley.edu

Table of Contents

Section S1. Select list of microdroplet reaction publications that contain “spontaneous” in the title.

Figure S1. Possible range of collision angles in the linear array detector.

Figure S2. Dependence of charge uncertainty on charge measurement.

Section S1. Select list of microdroplet reaction publications that contain "spontaneous" in the title

A reviewer questioned whether claims have been made in recent literature on microdroplet chemistry that unexpected reactions on microdroplet surfaces are spontaneous and not activated. We include a short but incomplete list of microdroplet chemistry papers where spontaneous reactions are reported and that include the word "spontaneous" in the title.

D. Zhang, X. Yuan, C. Gong, X. Zhang, High electric field on water microdroplets catalyzes spontaneous and ultrafast oxidative C–H/N–H cross-coupling. *J. Am. Chem. Soc.* **2022**, *144*, 16184–16190.

Yuan, X.; Zhang, D.; Liang, C.; Zhang, X. Spontaneous reduction of transition metal ions by one electron in water microdroplets and the atmospheric implications. *J. Am. Chem. Soc.* **2023**, *145*, 2800–2805.

Chen, H.; Wang, R.; Xu, J.; Yuan, X.; Zhang, D.; Zhu, Z.; Marshall, M.; Bowen, K.; Zhang, X., Spontaneous reduction by one electron on water microdroplets facilitates direct carboxylation with CO_2. *J. Am. Chem. Soc.* **2023**, *145*, 2647–2652.

Mehrgardi, M. A.; Mofidfar, M.; Zare, R. N. Sprayed water microdroplets are able to generate hydrogen peroxide spontaneously. *J. Am. Chem. Soc.* **2022**, *144*, 7606–7609.

Lee, J. K., Samanta, D.; Nam, H. G.; Zare, R. N. Micrometer-Sized Water Droplets Induce Spontaneous Reduction. *J. Am. Chem. Soc.* **2019**, *141*, 10585-10589.

He, J.; Zhao, Q.; Zhang, H.; Xu, Q.; Huang, J.; Shi, X.; Xia, D.; Hou, W.; Chen, J.; Jiang, J.; Xie, H.; Francisco, J. S. Spontaneous Reaction between CO_2 and Organic Acids in Water Microdroplets: Implications for the Formation of Secondary Organic Aerosols. *J. Am. Chem. Soc.* **2026**, *148*, 11240-11248.

Mofidfar, M.; Mehrgardi, M. A.; Zare, R. N. Water microdroplets surrounded by alcohol vapor cause spontaneous oxidation of alcohols to organic peroxides. *J. Am. Chem. Soc.* **2024**, *146*, 18498–18503.

Xia, D.; Zhang, H.; Ju, Y.; Xie, H.; Su, L.; Ma, F.; Jiang, J.; Chen, J.; Francisco, J. S. Spontaneous degradation of the "Forever Chemicals" perfluoroalkyl and polyfluoroalkyl substances (PFASs) on water droplet surfaces. *J. Am. Chem. Soc.* **2024**, *146*, 11266–11271.

Wang, X.; Han, J.; Diao, X.; Wang, X.; Zeng, C.; Li, Y.; Wang, J.; Cai, Z.; Wei, Z. Rational Control of Interfacial Spontaneous Redox Reactions by Modulating Single-Microdroplet Charging *Anal. Chem.* **2026**, *98*, 850–859.

Lee, K.; Mehrgardi, M. A.; Zare, R. N. Interfacial Curvature, not Simply Size, Controls Spontaneous Hydrogen Peroxide Formation in Water Microdroplets *J. A. Chem. Soc.* **2025**, *147*, 33240-33247.

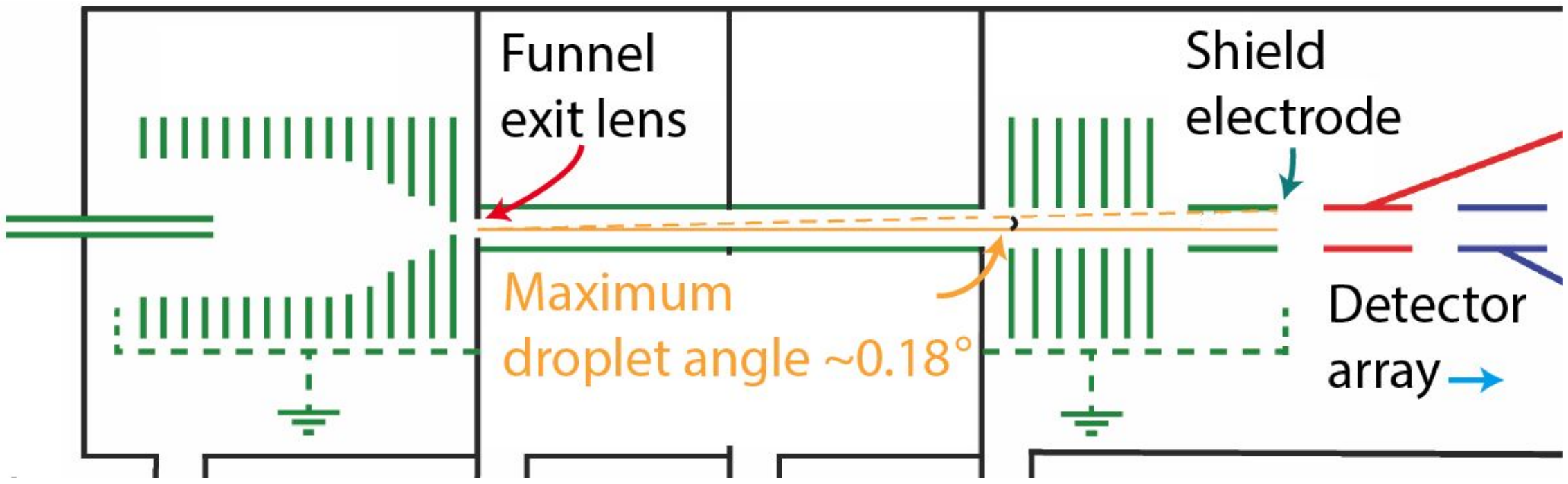

Figure S1: An expanded view of the Berkeley linear array charge detection mass spectrometer, annotated to illustrate the range of collision angles possible in the absence of aerodynamic turbulence past the high pressure (~4 Torr) ion funnel region. This indicates that incident droplets with an off-center angle of greater than ~0.18 degrees will collide with the shield electrode and not reach the detector.

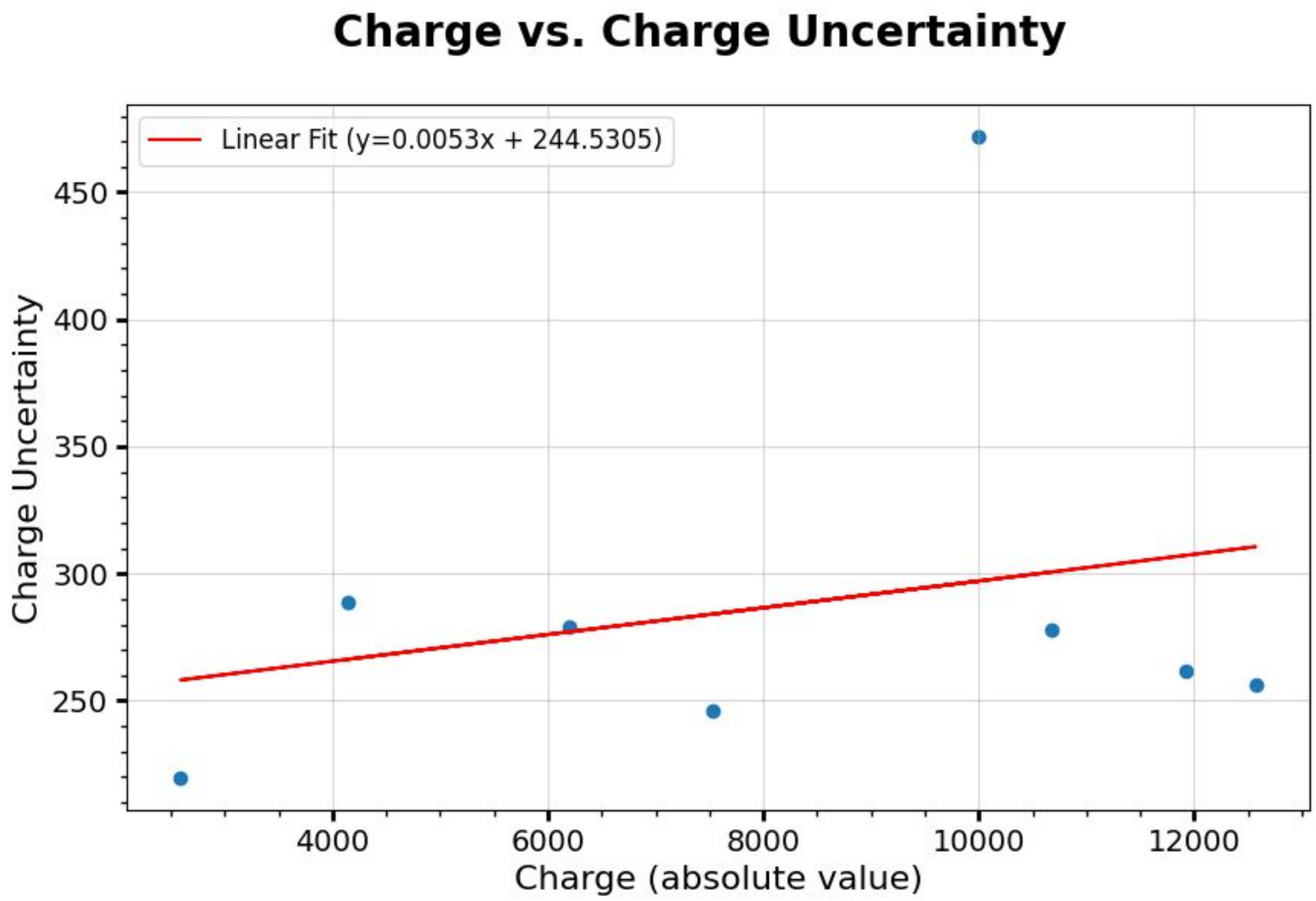

Figure S2: Charge uncertainty was measured for a series of 8 manually selected droplets that appear to pass cleanly through all 24 elements of the detector. Droplets range in charge from approximately –4,000 *e* to +13,000 *e*. Only the charge magnitude, which affects the amplifier response, is considered here. A slight trend towards higher uncertainty for larger droplets is observable. This is likely due to greater detector and amplifier 'settling' effects in the first 1 – 4 tubes that is more pronounced for larger droplets.
